# Ten days of supplementation with a standardized *Boswellia serrata* extract attenuates soreness and accelerates recovery after repeated bouts of downhill running in recreationally active men

**DOI:** 10.3389/fspor.2025.1488821

**Published:** 2025-01-23

**Authors:** Dawna Salter, Himana Yalamanchi, Aiswarya Yalamanchi, Amulya Yalamanchi

**Affiliations:** ^1^ Clinical Research and Innovation Division, PLT Health Solutions, Morristown, NJ, United States; ^2^ Department of General Medicine, Yalamanchi Hospital and Research Centre, Vijayawada, India

**Keywords:** DOMS, exercise-induced muscle damage, myalgia, inflammation, recovery, dietary supplement

## Abstract

Extracts derived from Indian frankincense (*Boswellia serrata)* effectively reduce the pain and swelling associated with osteoarthritis. It is unknown whether the anti-inflammatory and analgesic properties of *B. serrata* extend to muscle and joint pain resulting from high-impact eccentric exercise. This pilot study evaluated the efficacy of a standardized *B. serrata* extract LI51202F1 (SBS) to decrease soreness and improve recovery after repeated bouts of downhill running (DHR) compared to placebo (PLA). In total, 50 men (mean age 28 **±** 4) were randomly allocated to consume 60 mg SBS or PLA once per day for 10 days (6 days before, the day of, and 3 days after DHR). On day 7, delayed soreness was induced by three 15-min DHR episodes on a 10% declined treadmill. Visual analog scale (VAS) scores of joint and muscle soreness, in addition to the maximal weight lifted for a one-repetition leg extension (1RM-LE) were assessed at baseline (pre-supplementation) and on days 8, 9, and 10. Blood and urine samples collected at baseline, before, and after DHR were analyzed for inflammation-related biomarkers. VAS scores significantly increased while muscle strength decreased from baseline measures after DHR regardless of group assignment (main effect of time *p* < 0.001). However, the subjects who received the supplement reported reduced muscle soreness on days 8, 9, and 10 compared to those who received the PLA. On day 9, peak soreness scores were lower in the SBS group than in the PLA group (*p* = 0.001). On day 10, the SBS group’s VAS scores were 8.0 ± 1.6 mm lower than the PLA group (*p* < 0.001). Supplementation improved recovery time as the 1RM-LE returned to baseline strength by day 10, with the subjects who received the supplement lifting 3.4 ± 1.2 kg more than the PLA group (*p* = 0.006). The SBS-supplemented subjects reported less knee joint pain after DHR than the PLA group (*p* < 0.05 on days 8, 9, and 10). C-reactive protein and interleukin-6 increased after DHR, but by day 10, SBS reduced these markers compared to the PLA (*p* < 0.05). This study suggests that SBS, a novel, standardized extract derived from the gum resin of *B. serrata*, improves recovery and reduces soreness following high-impact eccentric exercise.

**Clinical Trial Registration:**

https://ctri.nic.in/Clinicaltrials/pubview2.php, Clinical Trial Registry of India (CTRI/2019/07/020323).

## Introduction

Excessive or unaccustomed physical activity can result in discomfort, stiffness, and a loss of strength (1). When the activity involves eccentric muscle contraction, the damage and inflammation can cause delayed onset muscle soreness (DOMS), a situation marked by sore, painful muscles not perceived until 24–48 h after the exercise was completed (2). Research has indicated that DOMS can take a week or more to fully resolve (1), causing training and performance disruptions for recreational or competitive athletes. DOMS can also affect less active and sedentary individuals after beginning a new form of exercise or simply overdoing repeated basic muscular movements. Thus, finding treatments to ameliorate soreness and help accelerate recovery would benefit all people who experience DOMS following unaccustomed exercise or activity.

A variety of modality, nutritional, supplemental, and pharmaceutical approaches have been explored to remediate DOMS and have been met with varying degrees of success (2–9). Non-steroidal anti-inflammatory drugs (NSAIDs) are frequently used to alleviate DOMS because they inhibit the production of pro-inflammatory prostaglandins. However, they may also negatively influence post-exercise muscle synthesis and delay the healing of damaged tissues through their selective inhibition of cyclo-oxygenase-II (COX-2) (2, 8, 10, 11). Therefore, there is interest in finding safe and effective alternative methods to attenuate inflammation, manage pain, and improve recovery after activities that result in DOMS.

The gum resin of the *Boswellia serrata* tree has long been used by traditional medical practitioners as an anti-inflammatory and analgesic agent (12, 13). *Boswellia* extracts standardized to the major active constituent of the gum resin, 3-O-acetyl-11-keto-*β*-boswellic acid (AKBA), inhibit 5-lipoxygenase (5-LOX), a key enzyme in the biosynthesis of inflammatory leukotrienes (13, 14). AKBA-enriched *Boswellia* extracts have been shown to be effective against inflammatory conditions and can improve the pain and swelling associated with osteoarthritis (13, 15). Previously, 100 mg of a synergistic extract derived from *B. serrata* gum resin standardized to 20% AKBA was shown in placebo-controlled studies to improve osteoarthritis symptoms and to normalize inflammatory biomarkers when consumed by subjects with mild to moderate knee osteoarthritis for 30 (16) and 180 days (17). Further, these osteoarthritic subjects reported significant pain relief in as few as 5 days after beginning supplementation (16). A similar *B. serrata* extract standardized to 30% AKBA was shown to improve function and relieve osteoarthritic pain in as few as 7 days of daily consumption (18). It is unknown whether AKBA-enriched *Boswellia* extracts might impact the inflammation, soreness, and functional impairments that follow eccentric exercise similar to what has been shown in research studies investigating other anti-inflammatory plant-based supplements, such as curcumin or tart cherries (19–24).

Standardized *Boswellia* supplement LI51202F1 (SBS) was designed as a water-soluble *B. serrata* gum resin extract standardized to contain a minimum of 76% AKBA. This study aimed to determine whether 10 days of supplementation with a 60 mg dose of SBS would impact subjective measures of muscle and joint soreness, muscle strength, and ratings of perceived exertion following repeated episodes of downhill running (DHR). DHR is a well-established eccentrically biased exercise that imparts repetitive high-impact mechanical loading to elicit DOMS and joint stress, as muscle and knee joint tissues are used to absorb the shock of the foot strike and brake against the downhill slope (25–27). In addition, biomarkers of inflammation and clinical safety parameters were assessed.

## Materials and methods

### Ethics approval and registration

This 10-day randomized, double-blinded, placebo-controlled study was conducted at an independent research organization according to the Declaration of Helsinki in agreement with the International Conference of Harmonization guidelines on Good Clinical Practice. The trial protocol was approved by the Yalamanchi Hospital and Research Center's Institutional Ethics Committee (Andhra Pradesh, India), and the study was registered with the Clinical Trial Registry (CRTI) of India (Registration no. CTRI/2019/07/020323).

### Study participants

Healthy, normal to overweight (body mass index between 20 and 29.9 kg/m^2^) men between 25 and 40 years, who were recreationally active for the previous 6 months were recruited for the study. Recreationally active was defined as 15–30 min of resistance exercise, and/or 30–60 min of low-to-moderate intensity aerobic activity, and/or 1–3 h of recreational sports participation per week. Men were excluded if they had knee pain or injury, had consumed analgesics or NSAIDs within 1 week of screening, or had utilized dietary supplements known to impact muscle recovery or performance in the previous month. Each volunteer was aware of the study protocol and provided written informed consent before any study-related procedures were conducted.

### Study design

In total, 50 subjects were enrolled and randomly allocated into one of two groups (*n* = 25) by following the randomization codes as generated by the SAS procedure PROC PLAN using a block design: supplement (SBS) or placebo (PLA). This study involved six visits to the study site in which the subjects arrived in the morning after 10 h of fasting: screening (day −7), randomization/baseline (day 1), DHR exercise day (day 7), 24-h post-exercise (day 8), 48-h post-exercise (day 9), and 72-h post-exercise (day 10). The subjects consumed their first dose at the study site on day 1 according to group assignment after all baseline assessments were concluded. Each subject was provided with six 60-mg sachet packages labeled with a randomization code with instructions to consume one sachet mixed with 200 ml of water once per day in the morning, on days 2 through 6. On day 7, urine and blood samples were collected, the study product was consumed with a light breakfast, and after a 30-min rest, the subjects performed the DHR exercise protocol. Immediately after completion of the DHR, blood and urine samples were collected for biomarker assessment. On days 7–10, after fasting, urine and blood samples were collected, the subjects consumed their assigned study product with a light breakfast. All physical assessments were conducted after 30 min.

**Figure 1:**
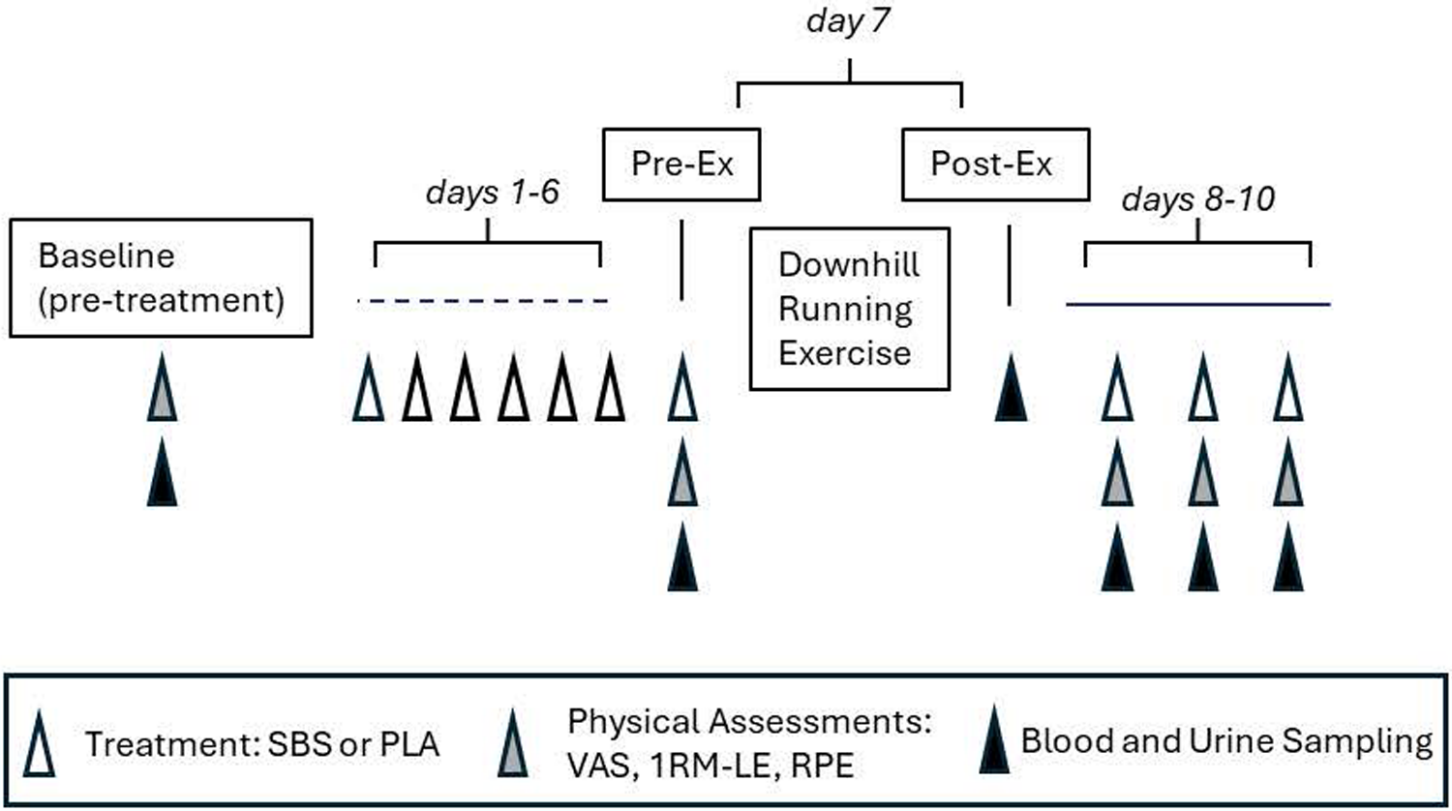
Schematic illustration of the experimental study design. Triangles illustrate the timing of treatment consumption and assessments during the study. Pre-Ex, immediately before the downhill running exercise protocol; Post-Ex, immediately after downhill running exercise protocol; SBS, standardized *Boswellia* supplement LI51202F1; PLA, placebo; VAS, visual analog scale; 1RM-LE, maximal weight lifted for one-repetition leg extension; RPE, rating of perceived exertion during 1RM-LE.

Supplement consumption compliance was monitored through daily diary recording and the collection/counting of all unused products. The subjects were instructed to maintain their routine diet and avoid strenuous exercise outside the study protocol. Adverse events, tolerance, and vital signs—heart rate, blood pressure, temperature, and respiratory rate—were assessed throughout the study and recorded at each study visit. Body weight was measured with a standard scale in triplicate to the nearest 0.1 kg, and the average was recorded at each visit. All participants, investigators, and study personnel remained blinded to group assignment throughout the study and until the data was locked. The study design is shown in Figure 1.

### Supplementation

The investigational product, SBS, was a water-soluble *B. serrata* extract standardized to a minimum of 76% AKBA. The investigational product and the placebo were manufactured by Laila Nutraceuticals (Vijayawada, India) under a strict Good Manufacturing Process. The products were matched in taste and appearance, packaged, and labeled with a randomization code. SBS is commercially available as AquaLOX™ and Dynagenix™ from PLT Health Solutions (Morristown, NJ, USA).

### Downhill running protocol

On day 7, all the subjects performed a DHR exercise protocol to induce muscular and joint stress. The subjects warmed up for 5 min by walking briskly at a self-selected pace on a level-grade treadmill (Spirit Fitness ST550 Treadmill) for 5 min, after which the treadmill was lowered to a decline of 10%. Each subject then ran for three episodes of 15 min, maintaining a heart rate at 80% of the calculated maximum. The heart rate was continuously monitored using pulse oximetry and was regulated manually by speed adjustments to the running belt to ensure the subject's performance was maintained at 80% of the calculated maximum. The heart rate was calculated using the equation of 208 − (0.7 × age) = maximal heart rate (28). Each downhill running episode was interspersed with 3–5 min of walking.

### Outcome measures

The primary endpoint evaluated was muscle soreness, measured using visual analog scale (VAS) scores while activating muscle contraction through squatting exercises at baseline (day 1) and for three days following the DHR exercise (days 8, 9, and 10). The secondary endpoints of muscle soreness while descending stairs, muscle strength, rating of perceived exertion (RPE) for a leg extension exercise, muscle stiffness, and knee joint soreness were also evaluated at baseline (day 1) and on days 8, 9, and 10. Biomarkers of inflammation and connective tissue damage—interleukin-6 (IL-6), high-sensitivity C-reactive protein (hs-CRP), and urinary cross-linked C-telopeptide of type II collagen (uCTX-II)—were assessed on day 1, on day 7 before and immediately after the DHR exercise, and once per day on days 8, 9, and 10. Cartilage oligomeric matrix protein (COMP) was measured on day 1 and twice on day 7, before and immediately after the DHR.

Safety parameters, including complete blood cell counts and blood chemistries— liver function tests, renal function tests, plasma total cholesterol, low- and high-density lipoprotein cholesterol, triglycerides, and fasting glucose— were assessed from fasting blood samples taken at screening and day 10. Test parameters for urine analysis included specific gravity, pH, albumin, bile salts, bile pigment, glucose, red blood cells, and ketones.

### Soreness and stiffness assessments

Muscle and knee soreness were quantified through each subject's self-assessment of pain while performing a squat exercise and again while descending stairs. For the squatting exercise, each subject performed two squats against their body weight where a depth was attained so that their upper legs were parallel (90°) to the floor while their arms were held horizontally in front of their shoulders. For the soreness assessment while descending stairs, each subject descended a 12-step staircase where each step measured 16 to 20 cm. Immediately following the squatting or descending-stair exercise, each subject marked the level of perceived pain on a 100 mm VAS in which 0 indicated “no pain” and 100 indicated “extreme pain” to represent the degree of soreness they experienced in the quadriceps femoris muscles. Overall muscle stiffness was evaluated with a 100 mm VAS in which 0 indicated “no stiffness” and 100 indicated “extreme stiffness” for the stiffness they felt in the quadriceps muscles.

### Muscle strength recovery and perceived exertion

Baseline muscle strength was determined by measuring each subject's maximal weight lifted for one repetition (1RM) in the leg extension exercise (LE, Viva Fitness Single Station Leg Extension/Leg Curl) on day 1 following the American Society of Exercise Physiologists’ procedure for the accurate assessment of muscular strength (29). Assessments were conducted under the supervision of a certified athletic trainer and trained study staff. Standardized instructions were provided on lifting techniques and testing procedures, standardized weights were utilized, and verbal encouragement was provided during testing. Subjects performed a warm-up series of 6 to 10 repetitions at approximately 50% of their estimated workload (predicted 1-RM) followed by a second series of 6 to 8 repetitions at 65% predicted 1-RM. Subjects performed a third series of two to four repetitions at 75% predicted workload, after which the subsequent lifts were single repetitions of progressively heavier weights until failure. The 1-RM was determined after three to five attempts at the heaviest single repetition weight, with a rest interval of 2 to 4 min between attempts. On days 8, 9, and 10 strength recovery was evaluated by measuring the maximal 1RM-LE the subject was able to complete in comparison to their baseline measures. The subjects’ RPE to complete a 1RM-LE was evaluated using the Borg Scale to rate their perceived exertion (30).

### Assessment of blood and urine parameters

Serum and urinary biomarkers were measured using standard enzyme immunoassay (ELISA) kits, following the manufacturer's instructions, including IL-6 (Diaclone SAS, Besancon Cedex, France), hs-CRP (Calbiotech, El Cajon, CA, USA), uCTX-II (Lifespan Biosciences, Seattle, WA, USA), and COMP (Ray Biotech, Peachtree Corners, GA, USA). The analytical sensitivities of the IL-6, hs-CRP, uCTX-II, and COMP ELISA kits were 2 pg/ml, 0.005 mg/L, 0.1 ng/ml, and 0.085 ng/ml, respectively.

Urine, serum, and whole blood samples were utilized for the safety and clinical assessments. Biochemical parameters were measured using a Cobas C 311 analyzer (Roche Diagnostics, Rotkreuz, Switzerland), and hematological parameters were measured using a Pentra ES 60 (Horiba ABX SAS, Montpellier, France). Urine analysis was conducted using standard Siemens Multistix 10 SG Reagent Strips (Siemens, Malvern, PA, USA) and by microscopy of the sediment.

### Statistical analysis

The data were analyzed using SPSS version 29.0 (SPSS, Inc., Chicago, IL, USA). The normality requirements of the population sample were evaluated using the Shapiro–Wilk test and histogram analysis. A mixed factorial analysis of variance (ANOVA) with repeated measures on time was conducted. The main effects (time or treatment) and interaction effect (time × treatment) were evaluated after the degrees of freedom were corrected by the Greenhouse–Geisser correction when the sphericity assumption was violated. To further clarify the magnitude of the main effects, the calculation of partial effect size (ɳ^2^) was used and was defined as small, ≥0.04 to <0.06; moderate, ≥0.06 to <0.14; and large, ≥0.14. When the F-ratio was significant for the main or interaction effects, *a priori* planned pairwise comparisons at each time point were conducted using paired (intragroup) or unpaired (intergroup) Student’s *t*-tests adjusted for multiple comparisons using the Bonferroni correction. The effects were considered significant at *p* < 0.05. Data are expressed as mean ± standard deviation (SD), and calculated mean differences (MD) are presented as means ± standard error of the measurement (SE) and reported in conjunction with the 95% confidence interval (CI).

**Figure 2:**
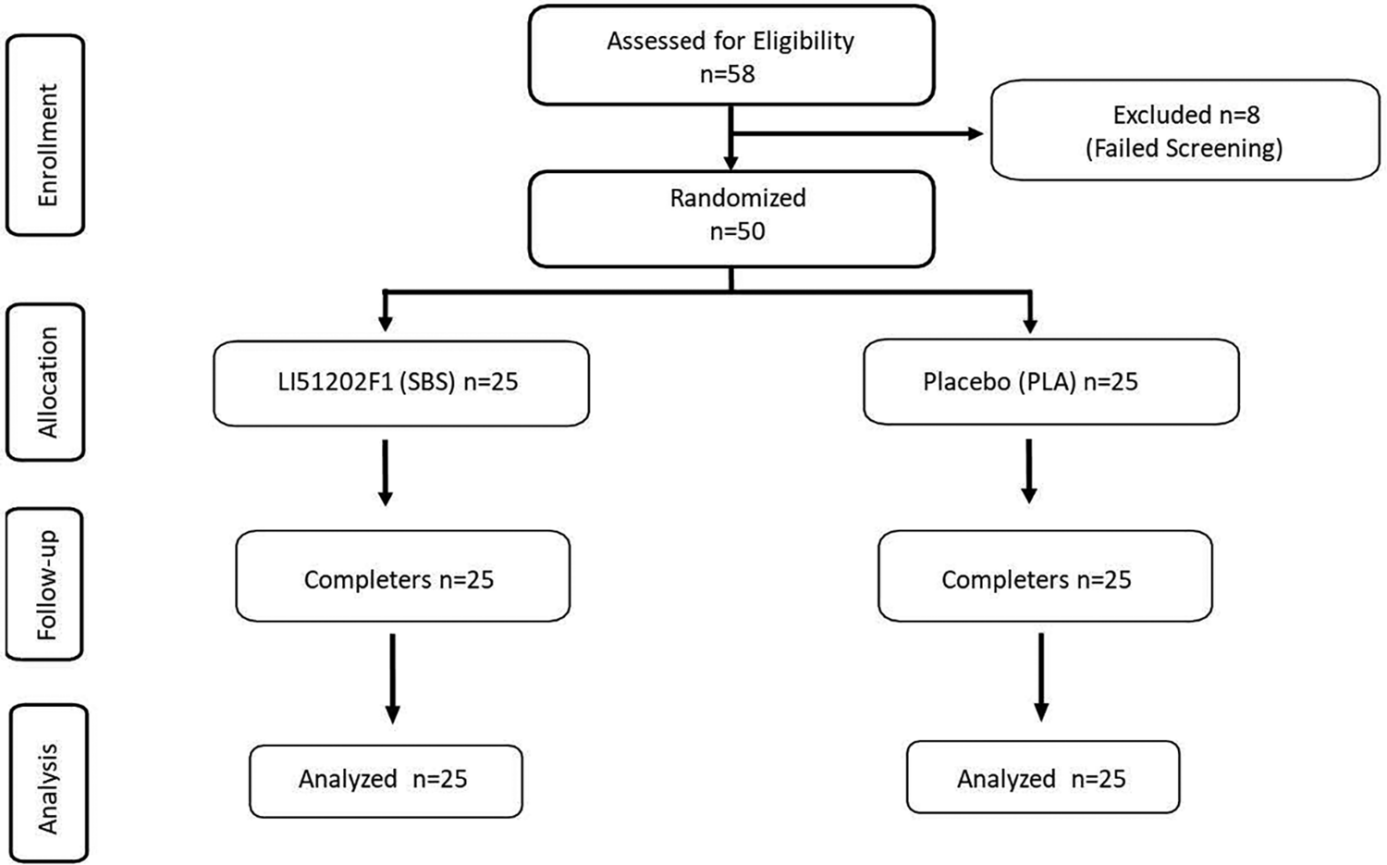
CONSORT diagram of the participant flow through the phases of the two-group parallel randomized blinded trial.

*A priori* analysis based on a clinical study conducted with a plant-based product (24) determined that a sample size of at least 23 subjects per treatment group would achieve 90% power to detect a treatment effect in the primary efficacy variable, VAS scores of muscle soreness, at a two-sided significance level of 0.05%. With an estimated 10% dropout rate, recruitment was determined at 50 subjects. In total, 58 subjects were assessed for eligibility, and eight failed screening due to not meeting the inclusion/exclusion criteria. Thus, 50 subjects were randomized into two equal groups (SBS or PLA, *n* = 25), and all the subjects completed the trial. The flow of the study progress is shown in the CONSORT diagram (Figure 2).

## Results

### Participant characteristics, adverse events, and compliance

The two groups were comparable in age, anthropometrics, and baseline measures before treatment began (Table 1). Compliance with treatment consumption was calculated at greater than 90% for all the subjects who completed the study. No serious adverse events were reported during the study or the post-study follow-up period. Overall, five subjects—three in the placebo and two in the treatment group— experienced minor adverse events. Specific minor adverse events in the supplemented group included one subject experiencing a minor cough, one reporting a headache, and one subject complaining of nausea. Adverse events within the placebo group consisted of one subject reporting nausea, one complaint of stomach pain, and one with a runny nose. These adverse events were self-limiting and resolved fully during the study. Safety parameters and vital signs were within normal clinical ranges at baseline and did not show significant changes in either subject group. Hematological and biochemical parameters are reported in Supplementary Tables S1 and S2.

**Table 1:** Participant characteristics at baseline.

Parameters	PLA	SBS	*p*-value
*N*	25	25	
Age (years)	28.4 **±** 4.2	28.4 ± 4.5	1.000
Height (m)	1.7 ± 0.1	1.7 ± 0.1	0.647
Weight (kg)	71.5 ± 7.2	71.5 ± 8.6	0.982
BMI (kg/m^2^)	26.1 ± 1.9	25.7 ± 1.9	0.481
VAS muscle soreness—squat (mm)	1.0 ± 2.0	0.80 ± 1.9	0.720
VAS muscle soreness—stairs (mm)	0.6 ± 1.7	0.4 ± 1.4	0.646

BMI, body mass index; VAS, visual analog scale; SBS, standardized *Boswellia* supplement LI51202F1; PLA, placebo.

Values are means ± standard deviation of the randomized population at study initiation.

### Muscle soreness

VAS scores of muscle soreness while completing a squatting exercise (MSSQ) and while descending stairs (MSDS) are presented in Table 2. The analysis demonstrated a significant main effect of time and treatment, and a time ×  treatment interaction with large to moderate effect sizes for MSSQ (time: *p* < 0.001, ɳ^2^ = 0.946; treatment: *p* < 0.001, ɳ^2^ = 0.267; interaction: *p* < 0.001, ɳ^2^ = 0.166) and MSDS (time: *p* < 0.001, ɳ^2^ = 0.895; treatment: *p* = 0.008, ɳ^2^ = 0.138; interaction: *p* = 0.017, ɳ^2^ = 0.076). Within-group comparisons indicated that both groups experienced significantly more MSSQ and MSDS at all measured time points compared to their respective baseline scores, peaking 48 h post-exercise (*p* < 0.001). However, between-group comparisons indicated the SBS supplementation significantly decreased MSSQ compared to the PLA on day 8 (*p* = 0.013), day 9 (*p* = 0.001), and day 10 (*p* < 0.001). The mean MSDS VAS score in the SBS group was significantly lower than in the PLA group on day 10 (*p* < 0.001).

**Table 2:** Effect, effect size, and within- and between-group comparisons of muscle soreness, strength, and stiffness.

Parameter	Group	Evaluation days				Effect *p*-value and effect size (ɳ^2^)
		Day 1 Baseline	Day 8 24 h after exercise	Day 9 48 h after exercise	Day 10 72 h after exercise	
VAS muscle soreness with squat (mm)	SBS ± SD	0.8 ± 1.9	28.0 ± 4.8^a^	31.0 ± 5.8^a^	9.0 ± 5.2^a^	(t) *p* < 0.001^c^ and ɳ^2^ = 0.946 (L)
	PLA ± SD	1.0 ± 2.0	32.0 ± 6.1^a^	36.4 ± 5.5^a^	17.0 ± 6.0^a^	(trt) *p* < 0.001^c^ and ɳ^2^ = 0.267 (L)
	MD ± SE	−0.2 ± 0.5	−4.0 ± 1.6^b^	−5.4 ± 1.6 ^b^	−8.0 ± 1.6 ^b^	(int) *p* < 0.001^c^ and ɳ^2^ = 0.166 (L)
	95% CI	−1.3 to 0.9	−7.1 to −0.9	−8.6 to −2.2	−11.2 to −4.8	
VAS muscle soreness descending stairs (mm)	SBS ± SD	0.4 ± 1.4	25.8 ± 5.7^a^	28.8 ± 7.5^a^	8.0 ± 4.8^a^	(t) *p* < 0.001^c^ and ɳ^2^ = 0.895 (L) (trt) *p* = 0.008^c^ and ɳ^2^ = 0.138 (M) (int) *p* = 0.017^c^ and ɳ^2^ = 0.076 (M)
	PLA ± SD	0.6 ± 1.7	29.4 ± 7.4^a^	32.4 ± 9.9^a^	15.0 ± 6.8^a^	
	MD ± SE 95% CI	−0.2 ± 0.4 −1.1 to 0.7	−3.6 ± 1.9 −7.4 to 0.2	−3.6 ± 2.5 −8.6 to 1.4	−7.0 ± 1.7 ^b^ −10.3 to −3.7	
1-mm leg extension (kg)	SBS ± SD	25.6 ± 4.2	21.0 ± 3.8^a^	19.0 ± 3.2^a^	26.4 ± 3.4	(t) *p* < 0.001^c^ and ɳ^2^ = 0.644 (L) (trt) *p* = 0.751 and ɳ^2^ = 0.002 (-) (int) *p* < 0.001^c^ and ɳ^2^ = 0.163 (L)
	PLA ± SD	26.6 ± 5.2	21.4 ± 5.3^a^	19.6 ± 4.8^a^	23.0 ± 4.8^a^	
	MD ± SE 95% CI	−1.0 ± 1.3 −3.6 to 1.7	−0.4 ± 1.3 −3.0 to 2.2	−0.6 ± 1.2 −2.9 to 1.7	−3.4 ± 1.2 ^b^ 1.0 to 5.8	
Rate of perceived exertion (score)	SBS ± SD	2.8 ± 1.4	4.6 ± 1.0^a^	5.2 ± 1.7^a^	3.0 ± 1.6	(t) *p* < 0.001^c^ and ɳ^2^ = 0.584 (L) (trt) p < 0.001^c^ and ɳ^2^ = 0.138 (M) (int) *p* = 0.081 and ɳ^2^ = 0.046 (S)
	PLA ± SD	3.0 ± 1.4	5.9 ± 1.1^a^	6.2 ± 1.1^a^	4.1 ± 1.3^a^	
	MD ± SE 95% CI	−0.2 ± 0.4 −1.0 to 0.6	−1.3 ± 0.3^b^ −1.9 to - 0.7	−1.0 ± 0.4^b^ −1.8 to −0.2	−1.1 ± 0.4^b^ −2.0 to −0.3	
VAS overall muscle stiffness (mm)	SBS ± SD	1.2 ± 2.2	28.2 ± 6.3^a^	33.6 ± 6.9^a^	9.4 ± 5.1^a^	(t) *p* < 0.001^c^ and ɳ^2^ = 0.911 (L) (trt) *p* = 0.002^c^ and ɳ^2^ = 0.182 (L) (int) *p* = 0.044^c^ and ɳ^2^ = 0.055 (S)
	PLA ± SD	1.4 ± 2.3	33.0 ± 8.9^a^	36.8 ± 6.1^a^	15.0 ± 5.4^a^	
	MD ± SE 95% CI	−0.2 ± 0.6 −1.5 to 1.1	−4.8 ± 2.2^b^ −9.1 to −0.5	−3.2 ± 1.8 −6.9 to 0.5	−5.6 ± 1.5^b^ −8.6 to −2.6	

Data are presented as mean ± SD or MD ± SE and 95% CI. Statistical significance was considered as *p* < 0.05 after the mixed factorial repeated measure ANOVA adjusted with the Bonferroni correction for multiple comparisons.

^a^ Within-group significance (vs. baseline).

^b^ Between-group significance (vs. placebo).

^c^ A significant main effect of time (t), treatment (trt), or the time × treatment interaction (int). Partial effect size (ɳ^2^) is defined as small, 0.04 (S); moderate, 0.06 (M); or large, 0.14 (L). SBS, *n* = 25; PLA, *n* = 25.

### Strength and perceived exertion for 1RM-LE

Muscle strength changes over time were measured by 1RM-LE and are presented in Table 2. Analysis of 1RM-LE demonstrated a main effect of time with a large effect size (*p* < 0.001, ɳ^2^ = 0.644) and an interaction between treatment and time with a moderately large effect size (*p* < 0.001, ɳ^2^ = 0.163). The main effect of the treatment was not significant (*p* = 0.751). Within-group comparisons illustrated that the PLA decreased leg strength compared to baseline on days 8, 9, and 10 (*p* < 0.001), while the mean 1RM-LE in the SBS group was significantly reduced from baseline on days 8 and 9 (*p* < 0.001), but not on day 10 (*p* = 1.00). The SBS-supplemented subjects recovered significantly more leg strength than those who received the PLA (26.4 ± 3.4 kg vs. 23.0 ± 4.8 kg, *p* = 0.006) on day 10 (Table 2). There was a significant main effect of time with a large effect size (*p* < 0.001, ɳ^2^ = 0.584) on the measures of RPE (Table 2), indicating the subjects in both groups perceived more effort with muscular work in the 3-day post-DHR recovery period. SBS supplementation also elicited a significant main effect of treatment with a large effect size (*p* < 0.001, ɳ^2^ = 0.206). There was no significant interaction between time and treatment, although a small effect size was noted (*p* < 0.081, ɳ^2^ = 0.055). Within-group comparisons illustrated that the PLA subjects significantly increased their mean RPE scores compared to baseline on days 8, 9, and 10 (*p* < 0.01). The RPE scores in the supplemented group were significantly increased on days 8 and 9 (*p* < 0.001) but recovered to baseline levels by day 10 (baseline score 2.8 ± 1.4 vs. day 10 score 3.0 ± 1.6, *p* = 1.00). Between-group comparisons illustrated that SBS supplementation significantly reduced RPE scores compared to the PLA on day 8 (*p* < 0.001), day 9 (*p* = 0.020), and day 10 (*p* = 0.009).

### Muscle stiffness

Measures of overall muscle stiffness as measured by a VAS are shown in Table 2. SBS affected muscle stiffness after the DHR as illustrated by the significant main effects of time and treatment with large effect sizes (*p* < 0.001, ɳ^2^ = 0.911; *p* = 0.022, ɳ^2^ = 0.182, respectively). There was also a time x treatment interaction with a small effect size (*p* = 0.044, ɳ^2^ = 0.055). Stiffness measures significantly increased in both groups compared to their baseline values at all measured time points (*p* < 0.001 on days 8, 9, and 10). Measures of stiffness were reduced with supplementation compared to the PLA on day 8 (*p* = 0.031) and day 10 (*p* < 0.001). On day 9, the muscle stiffness between-group comparison trended toward but did not reach significance (*p* = 0.088, Table 2).

### Knee joint soreness

The knee soreness VAS scores while completing a squatting exercise (KSSQ) and while descending stairs (KSDS) are presented in Table 3. The analysis demonstrated the main effects of time with large effect sizes for both KSSQ and KSDS (*p* < 0.001, ɳ^2^ = 0.903; *p* < 0.001, ɳ^2^ = 0.863, respectively) and significant main effects of treatment with large effect sizes (KSSQ, *p* = 0.005, ɳ^2^ = 0.156; KSDS, *p* = 0.002, ɳ^2^ = 0.180). Time × treatment interactions with small-to-moderate effect sizes were also shown (KSSQ, *p* = 0.049, ɳ^2^ = 0.057; KSDS, *p* < 0.001, ɳ^2^ = 0.123). Both groups experienced significantly (*p* < 0.001) more soreness on days 8, 9, and 10 for KSSQ and KSDS compared to their respective baseline scores (Table 3). However, between-group comparisons showed SBS supplementation significantly reduced KSSQ compared to the PLA at all measured time points (*p* = 0.013, *p* = 0.045, and *p* = 0.002 for days 8, 9, and 10, respectively). SBS supplementation also accelerated post-DHR recovery of KSDS with significantly reduced scores on both day 9 (*p* = 0.004) and day 10 (*p* < 0.001) compared to the PLA.

**Table 3:** Effects, effect size, and within- and between-group comparisons of knee soreness.

Parameter	Group	Evaluation days				Effect *P*-value and Effect Size (ɳ^2^)
		Day 1 Baseline	Day 8 24 h after exercise	Day 9 48 h after exercise	Day 10 72 h after exercise	
VAS knee soreness with squat (mm)	SBS ± SD	0.8 ± 1.9	26.2 ± 4.9^a^	30.4 ± 9.6^a^	10.6 ± 5.7^a^	(t) *p* < 0.001^c^ and ɳ^2^ = 0.903 (L) (trt) *p* = 0.005^c^ and ɳ^2^ = 0.156 (L) (int) *p* = 0.049^c^ and ɳ^2^ = 0.057 (S)
	PLA ± SD	1.0 ± 2.0	31.0 ± 7.9^a^	35.6 ± 8.2^a^	15.4 ± 4.6^a^	
	MD ± SE 95% CI	−0.2 ± 0.6 −1.3 to 0.9	−4.8 ± 1.9 ^b^ −8.5 to −1.1	−5.2 ± 2.5 ^b^ −10.3 to −0.1	−4.8 ± 1.5 ^b^ −7.7 to −1.9	
VAS knee soreness descending stairs (mm)	SBS ± SD	0.8 ± 1.9	25.0 ± 6.9^a^	23.0 ± 9.2^a^	6.2 ± 5.5^a^	(t) *p* < 0.001^c^ and ɳ^2^ = 0.863 (L) (trt) *p* = 0.002^c^ and ɳ^2^ = 0.180 (L) (int) *p* < 0.001^c^ and ɳ^2^ = 0.123 (M)
	PLA ± SD	0.8 ± 1.9	27.0 ± 8.0^a^	30.4 ± 7.9^a^	13.6 ± 5.5^a^	
	MD ± SE 95% CI	−0.0 ± 0.5 −1.1 to 1.1	−2.0 ± 2.1 −6.3 to 2.3	−7.4 ± 2.4 ^b^ −12.3 to −2.5	−7.4 ± 1.6 ^b^ −10.5 to −4.3	

Data are presented as mean ± SD or MD ± SE and 95% CI. Statistical significance was considered at *p* < 0.05 after the mixed factorial repeated measure ANOVA adjusted with the Bonferroni correction for multiple comparisons.

^a^ Within-group significance (vs. baseline).

^b^ Between-group significance (vs. placebo).

^c^ A significant main effect of time (t), treatment (trt), or the time x treatment interaction (int). Partial effect size (ɳ^2^) is defined as small, 0.04 (S); moderate, 0.06 (M); or large, 0.14 (L). SBS, *n* = 25; PLA, *n* = 25.

### Biomarkers of inflammation and tissue breakdown

**Figure 3:**
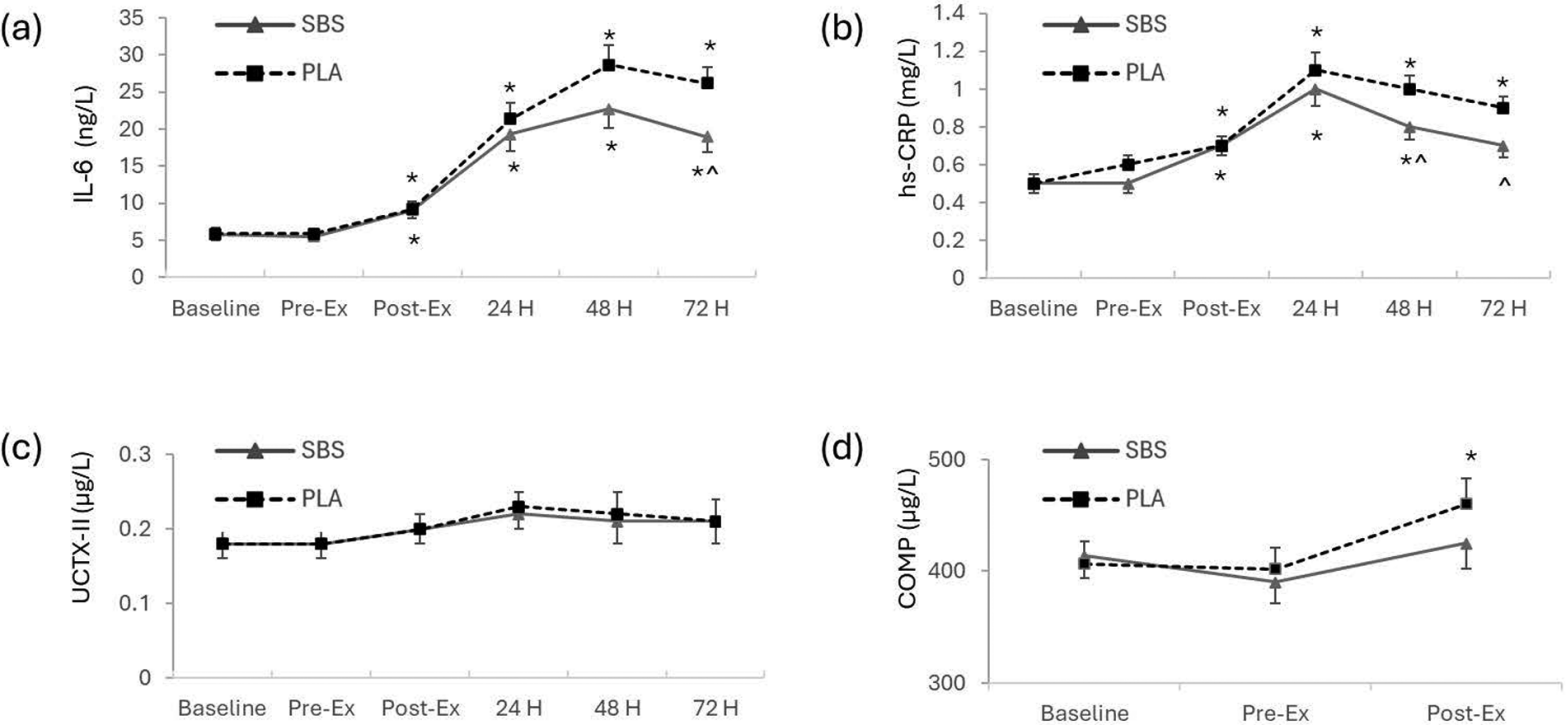
Changes in serum and urinary biomarkers. (**a**) IL-6, (**b**) hs-CRP, (**c**) uCTX-II, and (**d**) COMP. Data are presented as mean ± SE. Statistical significance was considered as *p* < 0.05 after the mixed factorial repeated measure ANOVA adjusted with the Bonferroni correction for multiple comparisons. *indicates within-group significance (vs. baseline) and ^ indicates between-group significance (vs. placebo). Pre-Ex, before downhill running protocol; Post-Ex, immediately after downhill running exercise protocol; 24H, 24 h after downhill running protocol; 48H, 48 h after downhill running protocol; 72H, 72 h after downhill running protocol; SBS, standardized *Boswellia* supplement L151202F1 group, *n* = 25; PLA, placebo group, *n* = 25.

IL-6 and hs-CRP significantly increased from baseline after downhill running, peaking between 24 and 48 h post-DHR exercise (Figure 3, Supplementary Table S3). Statistical analysis of IL-6 and hs-CRP demonstrated significant main effects of time with large effect sizes (IL-6, *p* < 0.001, ɳ^2^ = 0.654; hs-CRP, *p* < 0.001, ɳ^2^ = 0.557) but the main effect of treatment was not significant for either biomarker (IL-6, *p* = 0.139; hs-CRP, *p* = 0.279). The time × treatment interactions trended toward but did not attain significance, but small to moderate effect sizes were noted (IL-6, *p* = 0.066, ɳ^2^ = 0.054; CRP, *p* = 0.059, ɳ^2^ = 0.057). The mean serum values of IL-6 in the SBS group were significantly lower than the PLA group on day 10 (*p* = 0.021), whereas the SBS group's mean serum hs-CRP values were significantly lower than those of the PLA group on day 9 (*p* = 0.040) and day 10 (*p* = 0.037).

Analysis of the two biomarkers of cartilage metabolism, uCTX-II, and COMP demonstrated significant main effects of time and moderate effect sizes (*p* < 0.001, ɳ^2^ = 0.111; and *p* = 0.005, ɳ^2^ = 0.108, respectively), but no main treatment effects nor time x treatment interactions. Within-group comparisons indicated COMP values were significantly higher than baseline in the PLA group (*p* = 0.044) but not in the SBS group after completing the DHR exercise (Figure 3, Supplementary Table S3).

## Discussion

DHR is a high-impact exercise utilizing eccentric muscle activity and is well-documented to cause acute tissue injury and induce DOMS (25). This 10-day randomized trial demonstrated that the daily consumption of SBS before, during, and after repeated bouts of DHR ameliorated subjective measures of soreness, accelerated functional recovery, and decreased inflammation-related biomarkers compared to a placebo. SBS is a water-soluble extract derived from the gum resin of the *B. serrata* tree enriched to contain a minimum of 76% AKBA, a constituent compound with analgesic and anti-inflammatory properties (13, 14). In this study, subjects experienced the typical symptoms of DOMS, including sore, stiff muscles in the 24 to 48 h following DHR regardless of treatment group. However, those subjects randomized to receive daily SBS reported significantly less muscle soreness and overall stiffness compared to those who received the PLA, as indicated by VAS scores recorded over three recovery days. The VAS, in combination with a method of muscle stimulation such as squat exercises or descending stairs, has been widely used to quantify muscle soreness after exercise (19, 24, 31). The pain of DOMS is often accompanied by an inability to generate maximal muscular force (32). Indeed, all the subjects in the present study demonstrated this phenomenon 24 and 48 h after completing the repeated bouts of DHR, as their 1RM-LE weight was significantly reduced from their respective baseline measures. By day 10, the supplemented group recovered their baseline 1RM-LE strength, while those in the PLA group continued to show significant decrements. Alongside the reduction in muscular strength, both groups registered an increased mean RPE score when completing the 1RM-LE post-DHR. RPE is a validated marker of exercise intensity that enables the self-evaluation of how hard or easy muscular force or exertion feels at any given time through the integration of numerous internal homeostatic variables (33). Compared to the PLA group, the RPE scores were significantly reduced in the SBS group across the post-DHR 3-day recovery period, indicating that supplementation with this plant-based ingredient improved their perceived effort and the ability to generate muscular contraction after DHR-induced DOMS.

The reduction in muscular soreness and functional impairments following exercise due to SBS in this study is in line with effects shown by other natural plant-based compounds with known anti-inflammatory and analgesic properties (31, 34). Curcumin, a polyphenolic compound extracted from turmeric, has consistently been shown to help reduce muscle soreness after skeletal muscle damage (35). Curcumin at doses of 500 mg twice daily for 10 days (19) or 2,500 mg twice daily for 5 days (36) elicited reductions in lower limb pain over 3 days following unaccustomed eccentric leg exercises. While curcumin's overall hydrophobicity limits the gastrointestinal absorption of free curcumin (37), commercial methods exist to improve bioavailability and increase effectiveness with reduced dosages. Subjects provided twice daily 200 mg (21) or a once-daily 500 mg dose (20) of bioavailable curcumin formulations for 4 days reported less leg soreness 48 h after DHR. A single 500 mg dose of a bioavailable curcumin formulation, consumed 30 min before undergoing a lower limb eccentric muscle contraction protocol, was reported to reduce DOMS 48 and 72 h later (23). The Montmorency tart cherry is another natural product containing high levels of polyphenols that has been investigated for its efficacy in reducing DOMS following high-impact eccentric exercise. Ten days of daily supplementation of 480 mg of tart cherry powder was shown to decrease the perception of soreness 24 and 48 h after muscle-damaging exercise (22) and 10 days of twice daily 355 ml of tart cherry juice was shown to decrease the perception of muscle soreness after a long-distance relay race (24). Both curcumin and tart cherry are considered good sources of phenolic compounds with antioxidant and anti-inflammatory activity via modification of COX-2 enzyme pathways (38–40). Other plant-based nutrition interventions targeting DOMS, such as extracts derived from beets, quercetin, green tea, berries, or *Brassica*, have received attention, but evidence of their effectiveness remains preliminary (34). SBS is not rich in polyphenolic compounds but rather delivers a concentrated amount of AKBA (41), a naturally occurring pentacyclic triterpene shown to inhibit the production of leukotrienes (13, 14). Notably, at a relatively small daily dose of 60 mg, SBS significantly reduced perceived lower limb pain and accelerated the rate of muscle strength recovery in the days following DHR. By 72 h post-DHR, the mean soreness score on the 100 mm VAS was ∼50% lower with SBS than the PLA. The between-group total mean difference in the VAS scores was 8 mm for soreness while squatting and 7 mm for soreness when descending stairs; a substantial score difference when viewed in light of data suggesting a meaningful clinically important improvement for post-operative pain is a decrease of 10 mm (42).

Exercise overload, particularly that which involves the lengthening of the muscle while also using tension to brake against the direction of gravity, similar to that occurring in DHR, can result in subcellular micro-injuries within muscle fibers (25, 43), damage to fascial connective tissue (44, 45), increased reactive oxygen species, elevated cytokine release, and tissue inflammation (46, 47). Two serum markers that are elevated following strenuous DHR are hs-CRP and IL-6. Serum hs-CRP is an inflammatory marker shown to rise in situations of excessive exercise, joint injury, or longer-term joint inflammation (48–50). Compared to PLA, SBS supplementation significantly reduced elevated hs-CRP values by 20%–22% at 48 and 72 h after DHR. These data suggest that SBS may help attenuate the mild inflammatory response related to the muscle and joint impact following the high-impact DHR stimulus. IL-6 is a multifunctional molecule produced locally in skeletal muscle during exercise that is released acutely into the circulation at levels affected by exercise intensity and duration, the particular muscle groups exercised, and the nature of the muscular work completed (51–53). As such, running activates several large muscle groups and transiently elevates circulatory IL-6 levels during and immediately following the exercise. IL-6 can serve as both a pro- and anti-inflammatory cytokine and as a myokine involved in myogenesis (51, 54). IL-6 released from contracting muscle serves as a glucose sensor and metabolic mediator in an autocrine, paracrine, and endocrine fashion during exercise and elicits proliferation, differentiation, and fusion of satellite cells for muscle healing, regeneration, and training adaptations after exercise (53). However, muscle damage from eccentric muscle contraction (43, 46, 47); overtraining, particularly in untrained populations (52); and IL-6 release from peri-tendinous tissue responding to mechanical stress (55) can contribute to a prolonged elevation of circulatory IL-6, promoting pathways and receptor interactions involved in muscle wasting and exercise-induced or inflammatory pain (54, 56). Further, fast-twitch glycolytic muscle fibers are reported to be more vulnerable than slow-twitch oxidative fibers to damage from sustained elevated circulatory IL-6 levels (57), supporting the notion that a quick return of elevated circulatory IL-6 levels to basal levels is desirable to minimize exercise-induced muscle damage and inflammatory pain. In this study, the immediate increase of serum IL-6 relative to the baseline in both groups after DHR indicates that the molecule was likely released from the actively working skeletal muscle in a classic myokine-like fashion. Notably, this acute elevation was not affected by SBS. However, serum IL-6 continued to increase over the days following the DHR stimulus, peaking in both groups at the 48-h measured time point. The peak IL-6 value declined quicker with SBS than the PLA, so the mean values were 27% lower in the supplemented group by 72 h post-DHR. Thus, SBS supplementation did not attenuate the transient post-exercise elevations of circulatory IL-6 but did help normalize lingering elevated IL-6 levels. These observations suggest a unique specificity of SBS toward inhibiting potential deleterious inflammatory pathways but leaving intact those myokine pathways impacting muscle metabolism and recovery. Other anti-inflammatory compounds have been shown to impede muscle cell proliferation, muscle fiber growth, and repair through COX-2 enzyme inhibition (10, 11), whereas SBS, with its high AKBA content, likely imparts its benefits through non-competitive inhibition of 5-LOX, the enzyme that initiates the biosynthesis of inflammatory leukotrienes (14, 58). Notably, COX-1, and particularly COX-2, are lesser targets for AKBA (13), and preliminary preclinical research suggests the inhibition of the LOX pathways may support myogenesis and muscle regeneration after injury (59, 60). Nonetheless, the effects of extended use of SBS on skeletal muscle adaptation responses to moderate or intensive exercise regimens are unknown. Daily supplementation for as long as 180 days with an AKBA-enriched *B. serrata* gum resin extract has shown improved overall mobility and functionality with no safety concerns in osteoarthritic subjects (17). Research designed to investigate the effects of longer-term SBS supplementation on skeletal muscle adaptation to various exercise regimens should be considered.

SBS supplementation also impacted knee joint pain and function post-DHR. After DHR, the subjects in both groups reported increased knee joint soreness while squatting and descending stairs but those in the supplemented group reported significantly less joint pain compared to those in the PLA group. CTX-II, a marker associated with structural cartilage damage and type II collagen breakdown, and COMP, an extracellular matrix glycoprotein involved in cartilage breakdown or dysfunction (61, 62), are shown to increase immediately after dynamic, high-impact exercises such as downhill running (63, 64) in an intensity-dependent manner (48). In this study, the uCTX-II values did not change after DHR in either group. The COMP values were not significantly different between the groups after DHR, but COMP significantly increased compared with baseline values in the PLA group and this elevation was not measured in the SBS group. Growing evidence suggests that increased levels of biomarkers such as COMP following loading activities can provide a picture of how the joint tissue actively adapts to the loading stimulus and may serve as predictors of tissue structure change and overall joint health (48, 49). Research designed to further investigate the effects of SBS on joint metabolism, remodeling, and repair after or during high-impact activities may be warranted.

This study was limited by the fact that it only included male volunteers. Pain tolerance, muscle metabolism, and joint recovery can be sexually dimorphic (65–67), so future research that includes female subjects should be conducted. Another study limitation was the use of subjective assessment of pain after the DHR. While the VAS is well-validated for pain assessment (68), an additional measurement tool, such as a pressure algometer, could help quantify pain thresholds to a minimum stimulus intensity.

The findings of this study support the notion that supplementing SBS for 7 days before and 3 days after eccentrically biased exercise overload decreased measures of soreness and improved overall functional recovery. SBS supplementation was well-tolerated and effective against post-exercise-related muscle and joint pain in healthy volunteers, with no safety concerns. SBS mitigated muscle and joint soreness, accelerated functional recovery, and reduced deleterious inflammatory biomarkers following high-impact DHR. Individuals can overdo exercise without producing telltale warning signals of muscle damage, only to be surprised by substantial DOMS 24–48 h later. Lasting soreness and muscular dysfunction can markedly disrupt one’s ability to continue recreational or competitive sports, or even perform activities of daily living. SBS may offer protective effects if taken before and during episodes of physical activity involving excessive impact or eccentric muscular contractions.

## Data Availability

The original contributions presented in the study are included in the article/Supplementary Material, further inquiries can be directed to the corresponding author.
