# Supplementary material for: Ten days of supplementation with a standardized Boswellia serrata extract attenuates soreness and accelerates recovery after repeated bouts of downhill running in recreationally active men
Source: Front Sports Act Living. 2025 Jan 23;7:1488821. doi: 10.3389/fspor.2025.1488821 (PMC11798962; doi:10.3389/fspor.2025.1488821)
Supplement: Supplementary file 1 [file Table1.docx]

**Table S1: Hematologic parameters**

| Parameter | Evaluation  day | SBS  (n=25) | PLA  (n=25) |
| --- | --- | --- | --- |
|  |  |  |  |
| WBC  (cells/µL) | Screening | 7.9 ± 1.3 | 7.4 ± 1.7 |
|  | Day 10 | 7.4 ± 1.7 | 7.6 ± 1.8 |
| RBC (µL) | Screening | 5.4 ± 0.4 | 5.5 ± 0.4 |
|  | Day 10 | 5.1± 0.5 | 5.2 ± 0.5 |
| Hemoglobin  (g/dL) | Screening | 15.1 ± 1.0 | 15.3 ± 1.4 |
|  | Day 10 | 15.1 ± 1.3 | 15.0 ± 1.2 |
| Hematocrit  (%) | Screening | 47.4 ± 3.5 | 46.5 ± 2.9 |
|  | Day 10 | 47.0 ± 3.4 | 47.0 ± 2.4 |
| Platelet  (cells/µL) | Screening | 228.7 ± 58.9 | 242.9 ± 56.8 |
|  | Day 10 | 237.6 ± 56.8 | 240.9 ± 51.6 |
| Neutrophils  (%) | Screening | 50.5 ± 5.7 | 53.1 ± 7.5 |
|  | Day 10 | 52.8 ± 7.2 | 51.6 ± 7.7 |
| Lymphocytes  (%) | Screening | 39.4 ± 5.1 | 39.7 ± 4.7 |
|  | Day 10 | 37.5 ± 6.2 | 36.6 ± 7.2 |
| Monocytes  (%) | Screening | 6.4 ± 2.1 | 6.2 ± 2.1 |
|  | Day 10 | 7.0 ± 2.0 | 7.5 ± 1.1 |
| Eosinophils  (%) | Screening | 3.6 ± 2.6 | 3.7 ± 2.9 |
|  | Day 10 | 3.8 ± 2.7 | 3.0 ± 1.3 |
| Basophils  (%) | Screening | 0.22 ± 0.21 | 0.19 ± 0.15 |
|  | Day 10 | 0.14 ± 0.14 | 0.13 ± 0.11 |

Data presented as mean ± standard deviation of subjects who completed the course of study (n=50). WBC, white blood cells; RBC, red blood cells; SBS, Standardized Boswellia Supplement (LI51202F1); PLA, Placebo.

**Table S2: Serum biochemistry parameters**

| **Parameter** | **Day** | **SBS**  **(n=25)** | **PLA**  **(n=25)** |
| --- | --- | --- | --- |
|  |  |  |  |
| Fasting Glucose  (mg/dL) | Screening | 77.4 ± 11.7 | 81.0 ± 9.7 |
|  | Day 10 | 80.4 ± 9.2 | 77.1 ± 5.3 |
| Sodium  (mEq/L) | Screening | 140.9 ± 2.4 | 140.6 ± 2.1 |
|  | Day 10 | 141.8 ± 2.1 | 140.9 ± 2.3 |
| Potassium (mmol/L) | Screening | 4.0 ± 0.4 | 4.2 ± 0.4 |
|  | Day 10 | 4.1 ± 0.4 | 4.1 ± 0.4 |
| Urea Nitrogen (mg/dL) | Screening | 10.0 ± 2.4 | 11.2 ± 3.2 |
|  | Day 10 | 9.8 ± 2.1 | 9.9 ± 2.1 |
| Creatine  (mg/dL) | Screening | 0.9 ± 0.2 | 1.0 ± 0.2 |
|  | Day 10 | 0.9 ± 0.1 | 0.9 ± 0.2 |
| Albumin  (mg/dL) | Screening | 4.7 ± 0.3 | 4.9 ± 0.2 |
|  | Day 10 | 4.6 ± 0.3 | 4.7 ± 0.3 |
| Total Bilirubin (mg/dL) | Screening | 0.7± 0.4 | 0.6 ± 0.2 |
|  | Day 10 | 0.6 ± 0.2 | 0.7 ± 0.3 |
| ALP (U/L) | Screening | 91.2 ± 22.4 | 85.3 ± 23.7 |
|  | Day 10 | 83.2 ± 19.0 | 81.2 ± 20.9 |
| AST (U/L) | Screening | 23.0 ± 7.0 | 25.0 ± 6.1 |
|  | Day 10 | 22.6 ± 5.8 | 21.8 ± 4.7 |
| ALT (U/L) | Screening | 25.7 ± 11.4 | 27.2 ± 12.1 |
|  | Day 10 | 21.3 ± 7.9 | 20.2 ± 7.7 |
| T. cholesterol  (mg/dL) | Screening | 171.9 ± 29.8 | 147.7 ± 29.4 |
|  | Day 10 | 167.8 ± 30.4 | 158.3 ± 29.3 |
| Triglycerides (mg/dL) | Screening | 134.9 ± 93.4 | 124.9 ± 73.3 |
|  | Day 10 | 110.7 ± 44.8 | 117.6 ± 44.8 |
| HDL  (mg/dL) | Screening | 36.7 ± 5.5 | 39.8 ± 7.5 |
|  | Day 10 | 40.6 ± 12.8 | 41.1 ± 10.4 |
| LDL  (mg/dL) | Screening | 83.6 ± 20.3 | 106.4 ± 27.5 |
|  | Day 56 | 90.5 ± 22.5 | 99.5 ± 26.6 |

Data presented as mean ± standard deviation of subjects who completed the course of the study (n=50). T, Total; ALP, alkaline phosphatase; ALT, alanine transaminase; AST, aspartate aminotransferase; HDL, high-density lipoprotein; LDL, low-density lipoprotein; SBS, Standardized Boswellia Supplement( LI51202F1); PLA, Placebo.

**Table S3: Effects, effect size, within- and between-group comparisons of serum and urinary biomarkers.**

| Biomarker | Group | Evaluation days | | | | | | Effects P-value  Partial Effect Size ( ɳ^2^) |
| --- | --- | --- | --- | --- | --- | --- | --- | --- |
|  |  | Baseline  Day 1 | Day 7  before exercise | Day 7  after  exercise | Day 8  24h after exercise | Day 9  48h after exercise | Day 10  72h after exercise |  |
| IL-6 (ng/L) | SBS±SD | 5.8 ± 4.1 | 5.5 ± 3.4 | 9.1 ± 5.6* | 19.3 ± 11.0* | 22.7 ± 13.3* | 19.0 ± 10.0* | (t) P<0.001^#^  ɳ^2^=0.654 (L)  (trt) P=0.139 ɳ^2^=0.045 (S)  (int) P=0.066 ɳ^2^=0.054 (S) |
|  | PLA±SD | 5.9 ± 3.6 | 5.9 ± 3.4 | 9.2 ± 4.9* | 21.4 ± 11.2* | 28.7 ± 12.7* | 26.1 ± 11.0* |  |
|  | MD±SE  95% CI | -0.7 ± 1.1  -2.3 to 2.1 | -0.4 ± 1.0  -2.3 to 1.5 | -0.2 ± 1.5  -3.2 to 2.8 | -2.2 ± 3.1  -8.5 to -4.2 | -6.0 ± 3.7  -13.4 to 1.4 | -7.1 ± 3.0 ^  -13.1 to -1.1 |  |
| hs-CRP (mg/L) | SBS±SD | 0.53 ± 0.26 | 0.53 ± 0.25 | 0.72 ± 0.26* | 1.04 ± 0.49* | 0.78 ± 0.35* | 0.68 ± 0.30 | (t) P<0.001^#^  ɳ^2^=0.557 (L)  (trt) P=0.279 ɳ^2^=0.024 (-)  (int) P=0.059 ɳ^2^=0.057 (S) |
|  | PLA±SD | 0.52 ± 0.22 | 0.55 ± 0.26 | 0.74 ± 0.30* | 1.13 ± 0.44* | 0.98 ± 0.35* | 0.87 ± 0.32* |  |
|  | MD±SE  95% CI | -0.01± 0.07  -0.13 to 0.15 | -0.01 ± 0.07  -0.16 to 0.16 | -0.02 ± 0.08  -0.17 to 0.13 | -0.08 ± 0.13  -7.4 to 0.2 | -0.20 ± 0.10 ^  -0.40 to -0.10 | -0.19± 0.09 ^  -0.37 to -0.01 |  |
| uCTX-II (µg/L) | SBS±SD | 0.18 ± 0.11 | 0.18 ± 0.10 | 0.20 ± 0.11 | 0.22 ± 0.10 | 0.21 ± 0.11 | 0.21 ± 0.10 | (t) P<0.001^#^  ɳ^2^=0.111 (M)  (trt) P=0.952 ɳ^2^=0.000 (-)  (int) P=0.940 ɳ^2^=0.004 (-) |
|  | PLA±SD | 0.18 ± 0.12 | 0.18 ± 0.13 | 0.20 ± 0.11 | 0.23 ± 0.12 | 0.22 ± 0.14 | 0.21 ± 0.14 |  |
|  | MD±SE  95% CI | -0.00 ± 0.03  -0.06 to 0.07 | -0.00 ± 0.03  -0.06 to 0.07 | -0.00 ± 0.03  -0.06 to 0.07 | -0.01 ± 0.03  -0.07 to 0.05 | -0.01 ± 0.04  -0.06 to 0.08 | -0.00 ± 0.04  -0.07 to 0.07 |  |
| COMP  (µg/L) | SBS±SD | 413.7 ± 91.8 | 390.1 ± 92.7 | 424.9 ± 107.7 | n.d | n.d | n.d | (t) P=0.005^#^  ɳ^2^=0.108 (M)  (trt) P=0.576 ɳ^2^=0.007 (-)  (int) P=0.323 ɳ^2^=0.023 (-) |
|  | PLA±SD | 406.9 ± 103.4 | 402.2 ± 95.6 | 460.2 ± 118.0* | n.d | n.d | n.d |  |
|  | MD±SE  95% CI | -6.8 ± 27.7  -48.8 to 62.4 | -12.1 ± 26.6  -65.6 to 41.5 | -35.2 ± 32.0  -99.5 to 29.1 |  |  |  |  |

Data presented as mean ± standard deviation (SD) or mean difference (MD) ± standard error (SE) and 95% Confidence Interval (95% CI). Significance is considered p <0.05 after mixed factorial repeated measure ANOVA adjusted with Bonferroni correction for multiple comparisons. *Indicates within-group significance (vs. baseline), ^ indicates between-group significance (vs. placebo), # indicates significant main effect of time (t), treatment (trt), or time x treatment interaction (int). Partial effect size (ɳ2) is defined as small, 0.04 (S); moderate, 0.06 (M); and large, 0.14 (L). IL-6 (interleukin-6), hs-CRP (high-sensitivity C-reactive protein), uCTX-II (urinary cross-linked C-telopeptide of type II collagen), COMP (cartilage oligomeric matrix protein), SBS (Standardized Boswellia Supplement LI51202F1, n=25), PLA (Placebo, n=25), n.d. (not done at this time point).
